# Determination and Pharmacokinetics of Acetylcorynoline in Mouse Plasma by UPLC–MS/MS

**DOI:** 10.1155/ianc/5319104

**Published:** 2025-02-06

**Authors:** Mengzhi Xu, Xicheng Dong, Yishun Fan, Yucan Wang, Jinmiao Xu, Jianshe Ma, Xiaomin Yu

**Affiliations:** ^1^Department of Orthopedics, The First People's Hospital of Yuhang District, Hangzhou, China; ^2^Functional Experiment Teaching Center, School of Basic Medical Sciences, Wenzhou Medical University, Wenzhou, China; ^3^Department of Traditional Chinese Medicine, School of Traditional Chinese Medicine, Wenzhou Medical University, Wenzhou, China

**Keywords:** acetylcorynoline, bioavailability, mice, pharmacokinetics, plasma, UPLC–MS/MS

## Abstract

Acetylcorynoline is an alkaloid isolated from the tubers of *Corydalis ambigua* Cham. et Schltdl. It has anti-inflammatory properties with the potential to treat Parkinson's disease. However, the use of UPLC–MS/MS for identifying acetylcorynoline in mouse plasma has not yet been explored. The present study aimed to develop a fast and selective method for determining the amount of acetylcorynoline in mouse plasma using UPLC–MS/MS. Plasma samples (10 μL) were prepared using methanol-induced protein precipitation following the addition of aconitine as an internal standard. The chromatographic separation was accomplished using a UPLC HSS T3 column with acetonitrile and 0.1% formic acid as the mobile phase. The analytes were run for 4.0 min in total. The target fragment ions *m*/*z* 410.4 ⟶ 350.3 for acetylcorynoline and *m*/*z* 646.6 ⟶ 586.5 for internal standard were used for quantification using multiple reaction monitoring mode. The mouse blood was obtained at different time points after intravenous (5 mg/kg) and oral (20 mg/kg) administration of acetylcorynoline. The calibration plots for acetylcorynoline in mouse plasma showed a linear trend over the whole range of 1–2000 ng/mL. Both the intraday and interday precision relative standard deviations were less than 11%. The half-life in mice was found to be 2.6 ± 0.7 h and 2.7 ± 0.8 h following oral and intravenous administration, respectively. The bioavailability was determined to be 58.9%. The pharmacokinetics and bioavailability of acetylcorynoline in mice were effectively analyzed using this UPLC–MS/MS method, which had a runtime of 4 min per sample and required only 10 μL of plasma.

## 1. Introduction

Traditional Chinese medicines for pain relief include *Corydalis* species, which are well-known for their anti-inflammatory and anticancer properties [1, 2]. This species has been used in traditional Chinese medicine to treat various conditions, such as bleeding hemorrhoids, menstrual cramps, and nausea [3]. A prominent alkaloid derived from the tubers of *Corydalis ambigua* Cham. et Schltdl is acetylcorynoline. The anti-inflammatory properties of acetylcorynoline and its ability to reduce carbon tetrachloride (CCL_4_)-induced microsomal lipid peroxidation have been demonstrated in studies related to the treatment of Parkinson's disease (PD) [4, 5]. Acetylcorynoline has been shown to inhibit lipopolysaccharide-induced inflammation in breast tissue and human umbilical vein endothelial cells [6, 7].

The pharmacokinetics of acetylcarnitine has been less explored. Hence, a suitable method needs to be established for determining the concentration of acetylcarnitine in rat plasma and conducting pharmacokinetic studies. Data exist regarding the pharmacokinetic study and determination of acetylcorynoline concentration in rat plasma using LC–MS/MS. Wen et al. developed a liquid chromatography–mass spectrometry method for determining acetylcorynoline concentrations in rat plasma in the range of 5–1000 ng/mL to characterize the pharmacokinetic properties following the intravenous administration of 3 mg/kg acetylcorynoline [8]. The analysis of one sample required 100 μL of plasma and took 9 min. Wu and colleagues devised a highly sensitive method involving tandem mass spectrometry and liquid chromatography to simultaneously quantify nine different alkaloids, including acetylcorynoline, in human plasma [9]. The method required 600 μL of plasma and took 20 min to analyze a single sample, with a lower quantification limit of 15 pg/mL. However, the bioavailability of acetylcorynoline in mice or rats was not reported. Also, the use of UPLC–MS/MS to identify the pharmacokinetics and bioavailability of acetylcorynoline in mouse plasma has not been explored.

UPLC–MS/MS has several advantages over LC–MS/MS, such as quickness, high throughput, and solvent economy [10–15]. The UPLC–MS/MS method was used in this study to measure the pharmacokinetics and bioavailability of acetylcorynoline in mouse plasma, requiring a runtime of 4 min. The method worked well and only required 10 μL of plasma samples, making it suitable for use in examining the pharmacokinetics of acetylcorynoline.

## 2. Materials and Methods

### 2.1. Chemicals and Reagents

**Figure 1:**
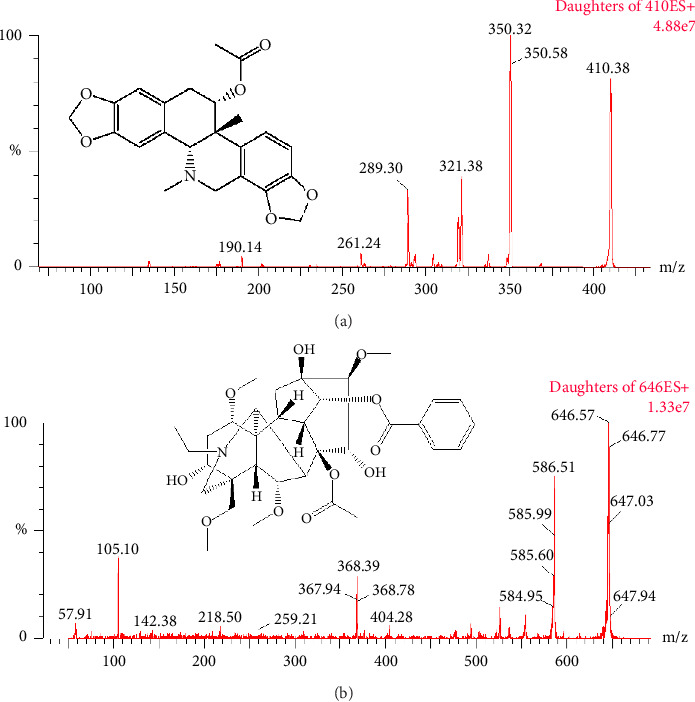
Mass spectrum of acetylcorynoline (a) and aconitine (IS, (b)).

Acetylcorynoline (purity > 98%, Figure 1(a)) and aconitine (IS, purity > 98%, Figure 1(b)) were purchased from Chengdu Mansite Pharmaceutical Co. Ltd. (Chengdu, China). Methanol and acetonitrile of chromatographic grade were acquired from Merck, Darmstadt, Germany. The Millipore Milli-Q purification system (MA, USA) produced ultrapure water. Mice devoid of drugs were used to obtain the blank plasma samples.

### 2.2. Instrumentation and Conditions

A UPLC–MS/MS system, including XEVO TQ-S micro triple quadrupole mass spectrometer and an ACQUITY H-Class UPLC system, was procured from Waters Corporation. An electrospray ionization (ESI) interface (Milford, MA, USA) was used for analyzing compounds. Software called MassLynx 4.1 (Waters Corp.) was employed for instrument control and data collection.

A UPLC HSS T3 (50 × 2.1 mm, 1.8 μm) column was used to separate acetylcorynoline and aconitine (IS). The column temperature was 40°C. The initial mobile phase comprised acetonitrile and water (containing 0.1% formic acid) with gradient elution at a flow rate of 0.4 mL/min. The percentage of acetonitrile ranged from 10% to 80% during the first 2 min, remained at 80% for 1 min, decreased from 80% to 10% in 0.5 min, and stayed at 10% for 1.5 min. The analytes were run for 4 min.

Employing a triple-quadrupole mass spectrometer fitted with an ESI interface in positive mode allowed for mass spectrometric detection. The gases used were 900 L/h nitrogen for desolvation and 50 L/h cone gas. The parameters used for ion monitoring were source temperature of 150°C, desolvation temperature of 450°C, and capillary voltage of 3.2 kV. The multiple reaction monitoring modes of *m*/*z* 410.4 ⟶ 350.3 for acetylcorynoline and *m*/*z* 646.6 ⟶ 586.5 for IS were used for quantitative analysis.

### 2.3. Calibration Standards

The stock solutions of aconitine (IS) (10.0 mg/mL) and acetylcorynoline (10.0 mg/mL) were made in a 50:50 (*v*/*v*) methanol–water mixture. Methanol was used to dilute the IS stock solution into a 50 ng/mL working standard solution. Similarly, stock solutions were used to create working solutions for calibration and controls. Before use, the temperature of all solutions was increased from 4°C (storage temperature) to room temperature.

Blank mouse plasma was spiked with appropriate concentrations of the working solutions to create calibration standards for acetylcorynoline. A short vortex mixing was performed after adding 10 μL of the suitable working solution to 100 μL of blank mouse plasma to offset calibration plots for acetylcorynoline in mouse plasma at concentrations of 1, 5, 10, 20, 50, 100, 200, 500, 1000, and 2000 ng/mL. Regarding the calibration standards, three distinct plasma concentrations (2, 180, and 1800 ng/mL) were prepared as quality control (QC) samples. Acetonitrile was used to precipitate the calibration standards and prepare the protein samples for UPLC–MS/MS analysis.

### 2.4. Sample Preparation

The plasma sample was thawed to room temperature before analysis. Then, 10 μL of the collected plasma sample was combined with an aliquot of 90 μL of the IS working solution (50 ng/mL) in a 1.5-mL centrifuge tube. The tubes were vortexed after 1.5 min. Further, 2 μL of the supernatant was introduced into the UPLC–MS/MS system for examination following a 10-min centrifugation at 14, 900*g*.

### 2.5. Method Validation

Extensive tests were performed in accordance with the standards established by the US Food and Drug Administration to fully validate the suggested bioanalytical method. The validation was conducted over 3 consecutive days. Each validation run involved six duplicates of QC plasma samples and one set of calibration standards.

#### 2.5.1. Selectivity

Six mouse plasma samples from different sources, along with LLOQ samples prepared using the corresponding mouse plasma, were analyzed using UPLC–MS/MS. This analysis assessed whether endogenous substances in blank plasma from different sources interfered with the determination of the target substances and internal standards.

#### 2.5.2. Calibration Curve

The weighted least square method (*W* = 1/*X*) was used to calculate the linear regression equation for 10 concentration levels of mouse plasma samples (1–2000 ng/mL) after sample preparation. The analyte concentration was plotted on the *x*-axis, and the peak area ratio of the analyte to the internal standard was plotted on the *y*-axis.

#### 2.5.3. Precision and Accuracy

The QC samples comprising low, medium, and high concentrations (2, 180, and 1800 ng/mL) of mouse plasma were taken. Six samples at each concentration were analyzed after sample processing and tested within 3 days. The concentration of each sample was calculated using the daily standard curve, and the intraday and interday precision and accuracy of this method were calculated based on the results of the QC samples [16].

#### 2.5.4. Matrix Effect

The supernatant was collected after treating blank plasma from six different sources, and the corresponding QC solutions with low, medium, and high concentrations of mouse plasma and internal standard solutions were added. The supernatant was analyzed using UPLC–MS/MS to obtain the corresponding peak area (A). At the same time, deionized water was used to replace mouse blank plasma, and the corresponding peak areas were obtained using the aforementioned method (B). The matrix effect was calculated using the ratio of the peak area of the two treatment methods for each concentration as follows: A/B × 100%. The matrix effect, normalized by the internal standard, was calculated as the ratio of the matrix effect of the analyte to that of the internal standard.

#### 2.5.5. Recovery

The samples prepared from low, medium, and high concentrations of blank mouse plasma were used. Six samples were analyzed for each concentration after sample processing. At the same time, the blank plasma of mice was treated with the corresponding low-, medium-, and high-concentration control solutions, and the supernatant was taken for analysis to obtain the corresponding peak areas (*n* = 3). The extraction recovery was calculated as the ratio of the chromatographic peak area after extraction to the peak area before extraction.

#### 2.5.6. Stability

The mouse plasma samples (2, 180, and 1800 ng/mL) were stored at room temperature for 6 h, the whole-plasma samples were stored at room temperature for 2 h, the precipitated protein samples were stored at room temperature for 6 h, and the autosampler was placed for 24 h. The samples were subjected to three freeze–thaw cycles at −20°C. The stability of mouse plasma samples was tested at −70°C for three freeze–thaw cycles.

#### 2.5.7. Robustness

The robustness was closely associated with intentional modifications in the developed method [17, 18]. The working solutions of acetylcorynoline at known concentrations were injected under varying conditions: the column oven temperature was adjusted to 37°C and 43°C, whereas the mobile phase flow rate ranged from 0.36 to 0.44 mL/min.

### 2.6. Pharmacokinetic Study

Twelve male mice weighing 20–22 g were procured from the Laboratory Animal Center of Wenzhou Medical University to investigate the pharmacokinetics of acetylcorynoline. The animal research was conducted in accordance with the guidelines established by the Animal Care and Use Committee of Wenzhou Medical University. The study protocol was approved by the Animal Care Committee of Wenzhou Medical University (wydw 2024-0232). Water was freely available, but diet was forbidden for 12 h prior to the experiment. The blood samples (30 μL) were drawn from the tail vein into heparinized 1.5-mL polythene tubes 0.08333, 0.5, 1, 2, 4, 6, 8, and 12 h following the intravenous (5 mg/kg) and oral (20 mg/kg) administration of acetylcorynoline. The samples were immediately centrifuged at 3000*g* for 10 min. The obtained plasma was kept frozen at −20°C until analysis. The plasma concentration of acetylcorynoline over time for each mouse was analyzed using the Drug and Statistics software (Version 2.0, Wenzhou Medical University).

## 3. Results and Discussion

### 3.1. Method Development

A methodological evaluation frequently involves selecting between positive and negative ionization modes for ESI [19]. The analyte is typically deprotonated in the negative ion mode of charging, whereas protonation is the usual method in the positive ion mode. The weakly basic drugs include acetylcorynoline, which is an alkaloid compound. Positive ionization mode works better for weakly basic drugs. Acetylcorynoline exhibited greater sensitivity in the positive ESI mode in this study. Regarding the quantitative accuracy of the procedure, the selection of an internal standard is crucial in bioanalytical methodology. Aconitine was chosen because its mass spectrometric ionization mode was similar to that of acetylcorynoline.

UPLC conditions were as strict as possible to limit endogenous interference from the analyte and IS. Acetonitrile, methanol, a 0.1% aqueous solution of formic acid, and an ammonium acetate solution were all tested as mobile phases. Consequently, satisfactory chromatographic peaks and retention times were achieved using gradient elution with acetonitrile and 0.1% formic acid aqueous solution as the mobile phase. Isometric elution is more suitable for simple samples or rapid analysis. Gradient elution usually provides better separation performance, especially for complex mixtures. However, it may also require longer analysis time and more complex mobile phase preparation. Therefore, gradient elution was used in this study.

UPLC–MS/MS is more sensitive and faster compared with conventional HPLC [20]. An individual plasma sample can be analyzed in just 4 min, potentially saving a significant amount of time and solvents. Furthermore, acetylcorynoline has a relatively low limit of quantification (1 ng/mL), and hence, it can be used to estimate lower plasma concentrations at the final sampling time.

### 3.2. Method Validation

#### 3.2.1. Selectivity and Matrix Effect

**Figure 2:**
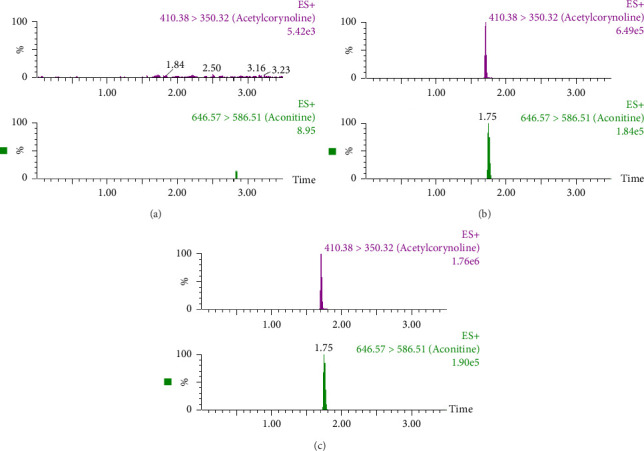
Representative UPLC–MS/MS chromatograms of acetylcorynoline and aconitine (IS). (a) Blank plasma spiked with IS. (b) Blank plasma spiked with acetylcorynoline and IS. (c) A mouse plasma sample after intravenous administration of a single dose of 5 mg/kg acetylcorynoline.

The standard chromatograms of a blank plasma sample, a blank plasma sample spiked with IS and acetylcorynoline, and a plasma sample are displayed in Figure 2. No endogenous substances were found to be interfering during acetylcorynoline retention.

The matrix effect for acetylcorynoline was between 90% and 107% (*n* = 6) at concentrations of 2, 180, and 1800 ng/mL. Therefore, the matrix effect from plasma was regarded as insignificant in the proposed method.

#### 3.2.2. Calibration Curve and Sensitivity

The linear regressions of the peak area ratios versus concentrations were fitted for a concentration range of 1–2000 ng/mL of acetylcorynoline in mouse plasma. The formula used to represent the calibration curve was *y* = 0.0249*x* + 0.0121 and *r* = 0.9988, where *x* is the plasma concentration and *y* is the ratio of the peak area of acetylcorynoline to that of IS. A measurement of 1 ng/mL acetylcorynoline in plasma was made using the LLOQ. The accuracy was 88.2%, and the precision was 14.5% at LLOQ.

#### 3.2.3. Precision, Accuracy, and Recovery

The relative standard deviation (RSD) for QCs at three concentration levels was calculated to assess the precision of the method after 3 days of validation testing (Table 1). The intraday precision was ≤ 8%, and the interday precision was ≤ 11% at every QC level. The accuracy of the method ranged from 91% to 112% at every QC level. The range of mean recoveries observed in mouse plasma for acetylcorynoline was 87%–94%.

#### 3.2.4. Stability

The autosampler results confirmed the analyte stability, which was within ±13% of the nominal values under room-temperature, freeze–thaw, and long-term (30-day) conditions, as shown in Table 2. Hence, the pharmacokinetic research could benefit from the established method.

#### 3.2.5. Robustness

The RSD was found to be < 8%, with minimal observed deviations. Consequently, the established method demonstrated robustness across diverse conditions, encompassing variations in column oven temperature and mobile phase flow rate. Therefore, the developed UPLC–MS/MS method exhibited remarkable efficiency.

### 3.3. Pharmacokinetic Study

**Figure 3:**
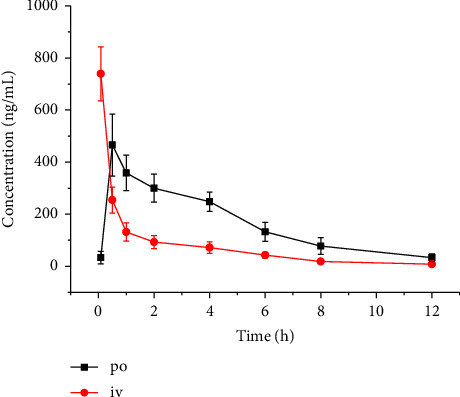
Mean plasma concentration–time profile after intravenous (5 mg/kg) and oral (20 mg/kg) administration of acetylcorynoline in mice.

The developed UPLC–MS/MS method was much faster than the reported LC–MS or LC–MS/MS method; it used only 10 μL of mouse plasma, with a runtime of 4 min [8, 9]. Mouse pharmacokinetic research was conducted using this UPLC–MS/MS method.  Figure 3 displays the mean plasma concentration–time curve following oral (20 mg/kg) and intravenous (5 mg/kg) acetylcorynoline administration. The pharmacokinetic curve showed no bimodal phenomenon, indicating that the intestinal drug absorption was complete after the oral administration of acetylcorynoline; no anorectal circulation was observed.  Table 3 provides an overview of the main pharmacokinetic parameters derived from the analysis of noncompartmental models.

Acetylcorynoline was absorbed quickly in mice (*T*_max_ was 0.6 ± 0.2 h). Its bioavailability was 58.9% in this study. The half-life (*t*_1/2_) of acetylcorynoline in mice was 2.6 ± 0.7 h and 2.7 ± 0.8 h following oral and intravenous administration, respectively. The *t*_1/2_ value of acetylcorynoline was 1.8 ± 0.1 h in rats following intravenous administration [8] but 4.13 h in human plasma following a single oral dose of Shuanghua Baihe tablet [9]. The *t*_1/2_ value was not the same after administering the Shuanghua Baihe tablet. Different administration methods (oral, sublingual, intravenous injection, etc.) can affect the absorption and distribution of drugs, thereby affecting their *t*_1/2_. Generally, the drugs administered intravenously tend to have a longer *t*_1/2_, whereas those administered orally or subcutaneously have a shorter *t*_1/2_. Additionally, there may be significant differences in drug metabolism and *t*_1/2_ between species such as mice, rats, and humans.

Most diseases require a multidose drug regimen to achieve effective treatment goals in clinical practice. Multidose administration, also known as repeated administration, refers to the method of administering drugs at a certain dose and interval, and with multiple repetitions to achieve and maintain a certain effective therapeutic blood concentration range. However, this study involved pharmacokinetic experiments on single-dose administration, which was also a limitation of our study.

## 4. Conclusions

This study developed a straightforward, exact, and accurate UPLC–MS/MS method for quantifying acetylcorynoline in mice with a runtime of 4 min for one sample using 10 μL of mouse plasma and an LLOQ of 1 ng/mL. Pharmacokinetic analysis of acetylcorynoline following intravenous and oral administration was effectively conducted using the UPLC–MS/MS method; the bioavailability was determined to be 58.9%. Acetylcorynoline can be used as an immunosuppressant; it can also reduce the damage to dopaminergic neurons in the brain of PD mouse model. The findings of this study on the pharmacokinetics and bioavailability of acetylcorynoline may provide a reference for the further development of acetylcorynoline-related drugs.

## Figures and Tables

**Table 1:** Accuracy, precision, recovery, and matrix effect of acetylcorynoline in mouse plasma (*n* = 6).

Concentration (ng/mL)	Accuracy (%)		Precision (RSD%)		Matrix effect (%)	Recovery (%)
	Intraday	Interday	Intraday	Interday		
8	98.6	91.2	4.5	2.7	90.8	93.8
180	105.0	95.9	3.8	10.7	94.5	93.0
1800	96.4	112.0	7.4	3.7	107.0	87.6

**Table 2:** Stability of acetylcorynoline in mouse plasma.

Concentration (ng/mL)	Autosampler (4°C, 12 h)		Ambient (2 h)		−20°C (30 days)		Freeze–thaw	
	Accuracy	RSD%	Accuracy	RSD%	Accuracy	RSD%	Accuracy	RSD%
8	104.0	1.6	107.3	8.6	107.5	3.3	96.5	6.9
180	96.3	3.9	99.2	4.9	102.5	11.5	90.7	3.3
1800	99.7	1.1	98.7	9.0	94.0	7.0	112.8	10.4

**Table 3:** Main pharmacokinetic parameters after administering acetylcorynoline in mice (*n* = 6).

Parameter	Unit	IV, 5 mg/kg	PO, 20 mg/kg
AUC_(0 − t)_	ng/(mL·h)	873.9 ± 132.8	2000.9 ± 320.8
AUC_(0 − *∞*)_	ng/(mL·h)	906.2 ± 132.8	2133.8 ± 397.1
*t*_1/2z_	h	2.6 ± 0.7	2.7 ± 0.8
*T*_max_	h	—	0.6 ± 0.2
CLz	L/(h·kg)	5.6 ± 0.9	9.6 ± 1.6
Vz	L/kg	21.3 ± 6.2	36.4 ± 8.9
*C*_max_	ng/mL	739.3 ± 103.3	470.1 ± 110.8

## Data Availability

The data supporting the findings of this study are available from the corresponding author upon reasonable request.
